# A Nucleotide Metabolite Controls Stress-Responsive Gene Expression and Plant Development

**DOI:** 10.1371/journal.pone.0026661

**Published:** 2011-10-19

**Authors:** Hao Chen, Baichen Zhang, Leslie M. Hicks, Liming Xiong

**Affiliations:** 1 Donald Danforth Plant Science Center, St Louis, Missouri, United States of America; 2 Division of Chemical and Life Sciences & Engineering, King Abdullah University of Science and Technology, Thuwal, Saudi Arabia; University of California, United States of America

## Abstract

Abiotic stress, such as drought and high salinity, activates a network of signaling cascades that lead to the expression of many stress-responsive genes in plants. The Arabidopsis FIERY1 (FRY1) protein is a negative regulator of stress and abscisic acid (ABA) signaling and exhibits both an inositol polyphosphatase and a 3′,5′-bisphosphate nucleotidase activity *in vitro*. The FRY1 nucleotidase degrades the sulfation byproduct 3′-phosphoadenosine-5′-phosphate (PAP), yet its *in vivo* functions and particularly its roles in stress gene regulation remain unclear. Here we developed a LC-MS/MS method to quantitatively measure PAP levels in plants and investigated the roles of this nucleotidase activity in stress response and plant development. It was found that PAP level was tightly controlled in plants and did not accumulate to any significant level either under normal conditions or under NaCl, LiCl, cold, or ABA treatments. In contrast, high levels of PAP were detected in multiple mutant alleles of FRY1 but not in mutants of other FRY1 family members, indicating that FRY1 is the major enzyme that hydrolyzes PAP *in vivo*. By genetically reducing PAP levels in *fry1* mutants either through overexpression of a yeast PAP nucleotidase or by generating a triple mutant of *fry1 apk1 apk2* that is defective in the biosynthesis of the PAP precursor 3′-phosphoadenosine-5′-phosphosulfate (PAPS), we demonstrated that the developmental defects and superinduction of stress-responsive genes in *fry1* mutants correlate with PAP accumulation *in planta*. We also found that the hypersensitive stress gene regulation in *fry1* requires ABH1 but not ABI1, two other negative regulators in ABA signaling pathways. Unlike in yeast, however, FRY1 overexpression in Arabidopsis could not enhance salt tolerance. Taken together, our results demonstrate that PAP is critical for stress gene regulation and plant development, yet the FRY1 nucleotidase that catabolizes PAP may not be an *in vivo* salt toxicity target in Arabidopsis.

## Introduction

Adverse environmental conditions such as drought, extreme temperatures, and high soil salinity can greatly reduce the yield of agricultural crop plants. Plants respond to these abiotic stresses by altering their physiology and development as well as by regulating an array of stress-responsive genes [1], [2], [3]. Collectively, the products of these genes can mitigate stress-caused damages or directly enhance plant tolerance to the stress. To understand how these stress-responsive genes are regulated, genetic screens using a luciferase reporter driven by a stress-responsive promoter were conducted in Arabidopsis [4], [5], and various mutants defective in expression of the reporter gene were isolated [4], [6]. One genetic locus defined in the study is *FIERY1 (FRY1)*, named for the high expression of stress-responsive luminescence in the mutants, which has been shown to play important roles in regulating gene expression and plant stress resistance [7].

*FRY1*, also known as *SAL1* [8], encodes a bifunctional enzyme with two distinct enzymatic activities: a nucleotidase activity that hydrolyzes 3′-phosphoadenosine-5′-phosphate (PAP) to AMP [9] and an inositol polyphosphate 1-phosphatase activity that catalyzes the breakdown of inositol polyphosphates, including the signaling molecule inositol 1,4,5-trisphosphate (IP_3_) [7], [8], [10]. *FRY1* null mutants and the *FRY1* low-temperature conditional mutant *hos2* showed a super induction of a reporter consisting of the ABA- and stress-responsive gene *RD29A* promoter-driven luciferase (*RD29A-LUC*) under multiple stress conditions or cold only [7], [10]. It was previously considered that the inositol phosphatase activity is responsible for repressing stress-responsive gene expression [7], [10], yet the contribution of the nucleotidase activity of FRY1 to stress gene regulation is unknown. Interestingly, in a separate genetic screen, FRY1 was identified as an RNA silencing suppressor related to its nucleotidase activity [11]. More recently, the FRY1 nucleotidase activity has also been suggested to be responsible for some other phenotypes of *fry1* mutants, such as its light sensitivity and reduced lateral root development [12], [13], [14], [15]. Nonetheless, in all previous studies the 3′,5′-bisphosphate nucleotidase activity of FRY1/SAL1 was assayed *in vitro*, but it is unknown whether this activity of FRY1 is essential for PAP degradation *in planta* and whether the activity is indeed responsible for some of the defects seen in *fry1* mutants. The major difficulty in addressing these questions is the lack of an accurate method to measure PAP levels in plants.

In this study, we developed a liquid chromatography coupled with tandem mass spectrometry (LC-MS/MS) method to detect and measure PAP so that its levels in plants can now be quantified. Using this method, we determined the PAP levels in wild type and *fry1* mutant plants subjected to NaCl, LiCl, low temperature, and ABA treatments. The possible role of PAP in regulating plant development and the *RD29A-LUC* reporter expression in response to stress in *fry1* mutants was also investigated by genetically eliminating PAP accumulation in the mutants. The function of FRY1 in salt stress tolerance was further studied with transgenic plants overexpressing the FRY1 protein. Our results indicate that the 3′, 5′-bisphosphate nucleotidase activity of FRY1 may not be an *in vivo* target of salt toxicity in Arabidopsis as it is in yeast, but that PAP accumulation is important for the superinduction of the *RD29A-LUC* gene by stress in *fry1* mutants.

## Results

### PAP content in seedlings under salt or cold stress or ABA treatment

**Figure 1:**
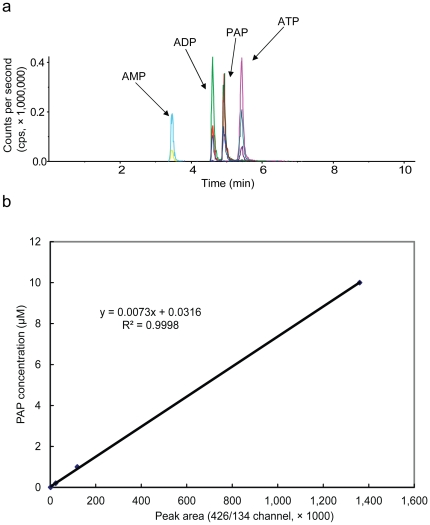
LC-MS/MS analysis of PAP, AMP, ADP, and ATP. (a) LC-MS/MS separation of PAP, AMP, ADP, and ATP. Two nmol each were used for the analysis. Different color lines represent different MRM transitions for each compound. For AMP, MRM transitions are 346/79 (top peak) and 346/134 (bottom peak). For ADP and PAP, peaks from top to bottom represent transitions 426/79, 426/134 and 426/348, respectively. For ATP, peaks from top to bottom represent transitions 506/159, 506/408 and 506/273, respectively. The unit for Y-axis is counts per second (cps). (b) Standard curve for PAP with LC-MS/MS method based on MRM detection. The standard curve was constructed with a serial dilution of a PAP standard, ranging from 4 nM to 50 mM. The MRM channel 426/134 was used for peak area integration.

While the nucleotidase activity of FRY1 was demonstrated with *in vitro* experiments, the activity has not been previously shown *in planta* due to the difficulty of measuring the substrate PAP in plant extracts. In this study, we developed an LC-MS/MS method to detect and quantify PAP in Arabidopsis seedlings. To maximize the sensitivity, a PAP standard was first used to optimize mass spectrometric parameters in the negative mode. PAP eluted after ADP and before ATP with baseline resolution. Three MRM transitions were chosen to increase the specificity and to distinguish PAP from its positional isomers by both the ratios of three MRM transitions and their different retention times (see Table 1 for MRM parameters). The ratio of MRM transitions 426/134 to 426/79 equals one for PAP, in contrast to 0.5 for ADP (Figure 1a). Using this method, slight retention time shifts and ATP ion source fragments will not confound the appropriate assignment of PAP.

**Table 1:** MRM transitions for AMP, ADP, ATP, and PAP.

Compound	Q1	Q3	Dwell Time (ms)	DP (V)	EP (V)	CE (V)	CXP (V)
PAP/ADP	426	79	25	-72	-10	-69	-5
PAP/ADP	426	134	25	-72	-10	-40	-5
PAP/ADP	426	328	25	-72	-10	-26	-5
AMP	346	79	25	-75	-10	-55	-4
AMP	346	134	25	-75	-10	-47	-4
ATP	506	159	25	-80	-10	-42	-12
ATP	506	408	25	-80	-10	-32	-12
ATP	506	273	25	-80	-10	-42	-12

DP, declustering potential; EP, entrance potential; CE, collision energy; CXP, collision cell exit potential.

The rapid degradation of nucleotides during extraction and ion suppression effect from co-eluting matrices pose special difficulties on analyzing nucleotides such as PAP. Fortunately, it was recently found that although water in extraction solvents leads to a rapid degradation of nucleotide triphosphates; an acidic environment could help to minimize the degradation [16]. Based on these new observations and the Fiehn protocol for plant metabolomics [17], we designed a rapid and reliable sample processing method in this study. Briefly, frozen plant tissues were ground to powder and extracted with a −20°C cold solvent mixture (chloroform:methanol:acetonitrile at 2∶1∶1 v/v/v with 0.4% formic acid). After quickly partitioning the organic extracts with ice-cold water, lipid and non-polar to medium-polar interfering compounds were removed, and the polar fraction was collected for analysis.

The LC separation and MRM-based detection method is very sensitive. A signal to noise (S/N) ratio above 3 was achieved for the lowest PAP concentration examined, 4 nM, which corresponds to a 40 femtomole on-column injection. A standard curve (Figure 1b) was constructed using a standard mixture of PAP, AMP, ADP, and ATP, covering 3 orders of magnitude (4 nM to 10 µM; for PAP, r^2^ = 0.9998). Analytical variability was checked with repeat injection of the same biological extracts containing PAP, with a resulting RSD of 15.98% (n = 3) for MRM channel 426/134. As low as 1 mg fresh weight equivalent Arabidopsis extract was sufficient for measuring all analytical target compounds (PAP, AMP, ADP and ATP). The latter three were also used for analytical quality control purposes. The low volume injection on the LC column also aids in minimizing the potential ion suppression commonly observed in LC-ESI-MS/MS analysis.

**Figure 2:**
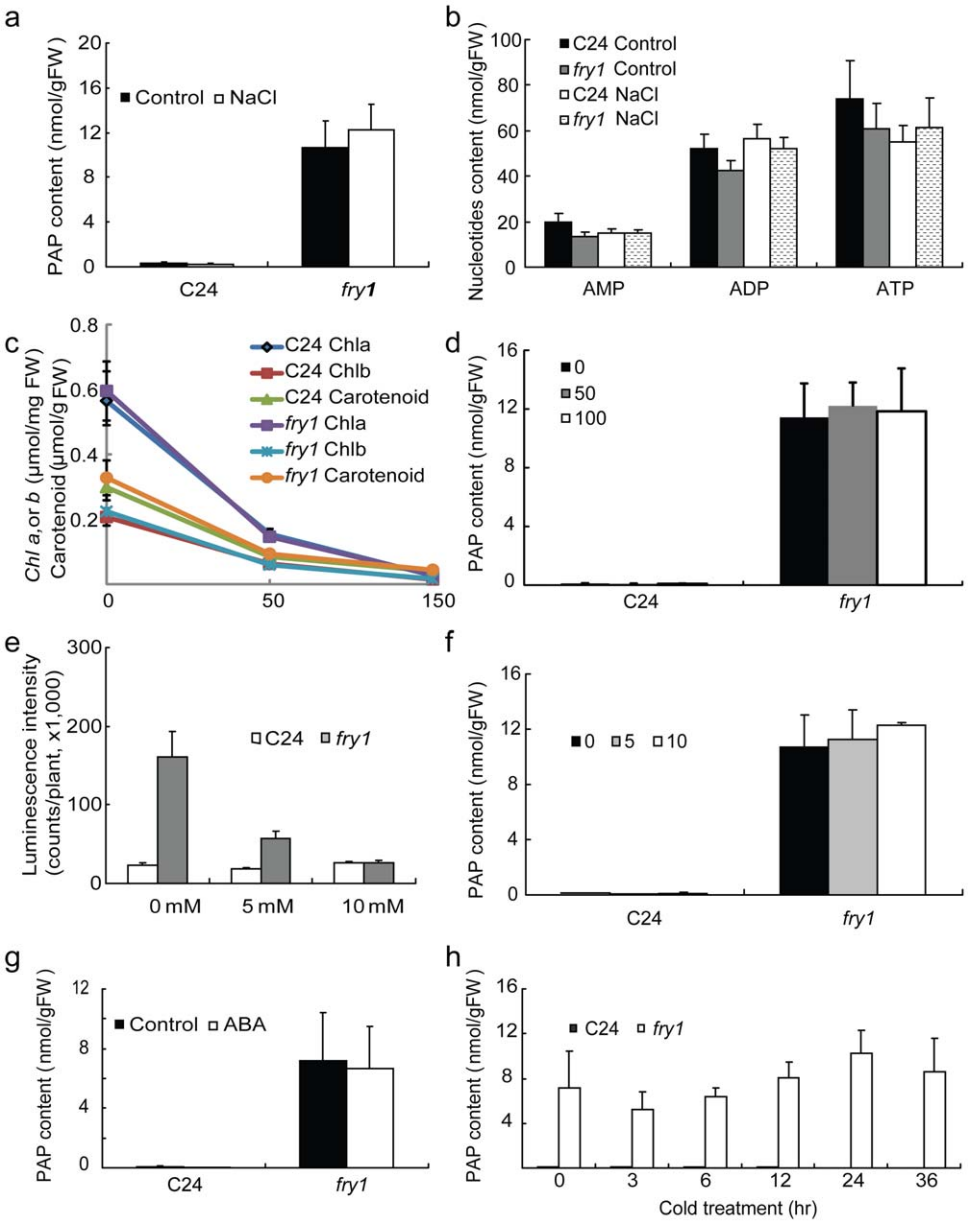
PAP content in wild-type C24 and *fry1* seedlings treated with either salt, ABA, or cold. (a) PAP content of seedlings treated with or without 300 mM NaCl for three hours. (b) AMP, ADP, and ATP content in seedlings treated with or without 300 mM NaCl for three hours. (c) Chlorophyll *a*, *b*, and carotenoid content in two-week-old wild-type C24 or *fry1* mutant seedlings grown on MS media supplemented with 0, 50 or 100 mM NaCl. (d) PAP content in two-week-old wild-type C24 or *fry1* mutant seedlings grown on MS media supplemented with 0, 50 or 100 mM NaCl. Data are means and standard errors from three biological replicates. (e) Luminescence intensity of cold-treated seedlings grown on MS media with or without supplement of the indicated concentrations of LiCl for two weeks. Cold treatment was conducted by incubating the seedlings at 0°C for 2 days before luminescence imaging. Luminescence expression was nearly undetectable before cold treatment (not shown). Data are means and SE from 30 seedlings. (f) PAP content in two-week-old seedlings grown on MS media supplemented with the indicated concentrations of LiCl. (g) PAP content of seedlings treated with 100 µM ABA for three hours. (h) PAP content of seedlings that were incubated at 0°C for the indicated time period. Data are means and SE from three biological replicates. Note that in (a), (d), (f), (g), and (h), PAP levels in wild-type C24 seedlings are very low.

AMP, ADP and ATP are structurally similar with PAP. As isomers, PAP and ADP were closely co-eluted, yet they were base-line resolved in our method (Figure 1a). Given that no stable isotope-labeled standard was available for PAP, the reliability of the method was first established by comparing the measured AMP, ADP and ATP levels with reported levels obtained with other traditional methods, such as enzymatic coupled assay [18] or spectroscopic methods [19]. Despite the slight difference in plant tissue types in this study and those of others, all of the AMP, ADP and ATP levels measured in this study were similar to those measured by traditional methods. For example, around 70 nmol/g FW (fresh weight) for ATP and around 40 nmol/g FW for ADP were found in our study (Figure 2b), similar to the levels reported using other methods [18], [19]. These comparisons reveal that ion suppression is not a major issue for our method. This was further confirmed by spiking a known concentration of PAP standard into Arabidopsis leaf extracts, with no significant ion suppression observed (data not shown).

Using this method, we measured the PAP content in Arabidopsis seedlings. PAP levels in wild-type seedlings grown on the Murashige and Skoog (MS) medium were barely detectable. In contrast, the *fry1-1* null mutant [7] (referred to as *fry1* hereafter) grown under the same conditions accumulated over 10 nmol PAP per gram fresh weight (Figure 2a). Treatment with 300 mM NaCl for 3 hours did not have any significant effect on PAP accumulation in either the wild type or *fry1* (Figure 2a). The accumulation of PAP in *fry1* is specific, since the levels of the other adenine nucleotides AMP, ADP, and ATP in *fry1* did not have dramatic changes relative to those in the wild type (Figure 2b). The levels of these nucleotides also did not change after the NaCl treatment. To determine whether longer salt stress treatment would affect PAP accumulation, we directly planted the wild type and *fry1* seeds on MS media supplemented with or without 50 or 100 mM NaCl. These salt treatments caused clear stress to the seedlings, as indicated by yellowish leaves that can also be seen with greatly decreased chlorophyll *a*, *b*, and carotenoid levels as salt concentrations increased (Figure 2c). Under these conditions, no significant increase in PAP was observed in either the wild type or *fry1* seedlings (Figure 2d).

Previous *in vitro* studies suggest that FRY1 and its homologs are more strongly inhibited by lithium than by sodium [10], [20], yet whether FRY1 is similarly inhibited *in planta* is unclear. We previously found that LiCl treatments of the wild-type seedlings (containing the *RD29A*-*LUC* gene) for ten hours did not result in enhanced induction of the *RD29A-LUC* reporter gene [10], as would be expected if the FRY1 enzyme were a target of Li toxicity and inhibited by the treatments. Here we treated seedlings with LiCl for an extended period of time (two weeks), yet we still did not see the hyperinduction of the *RD29A*-*LUC* gene by cold (Figure 2e). To further determine whether the enzymatic activity of FRY1 was inhibited *in planta*, we checked whether PAP accumulated under Li treatments. Seedlings were allowed to grow on MS media supplemented with different concentrations of LiCl for two weeks before samples were extracted for PAP measurements. It was found that Li treatments did not lead to more PAP accumulation compared with seedlings grown on the control conditions without Li (Figure 2f).

Given the superinduction of the *RD29A*-*LUC* gene in *fry1* mutants upon treatment with different stresses including salt, ABA, and cold [7], we investigated whether this superinduction of the reporter gene in *fry1* has anything to do with increased PAP accumulation under these conditions. Similar to salt treatments, neither ABA nor cold treatments led to detectable increases in PAP accumulation in the wild type or to appreciable increases in *fry1* (Figures 2g and 2h).

**Figure 3:**
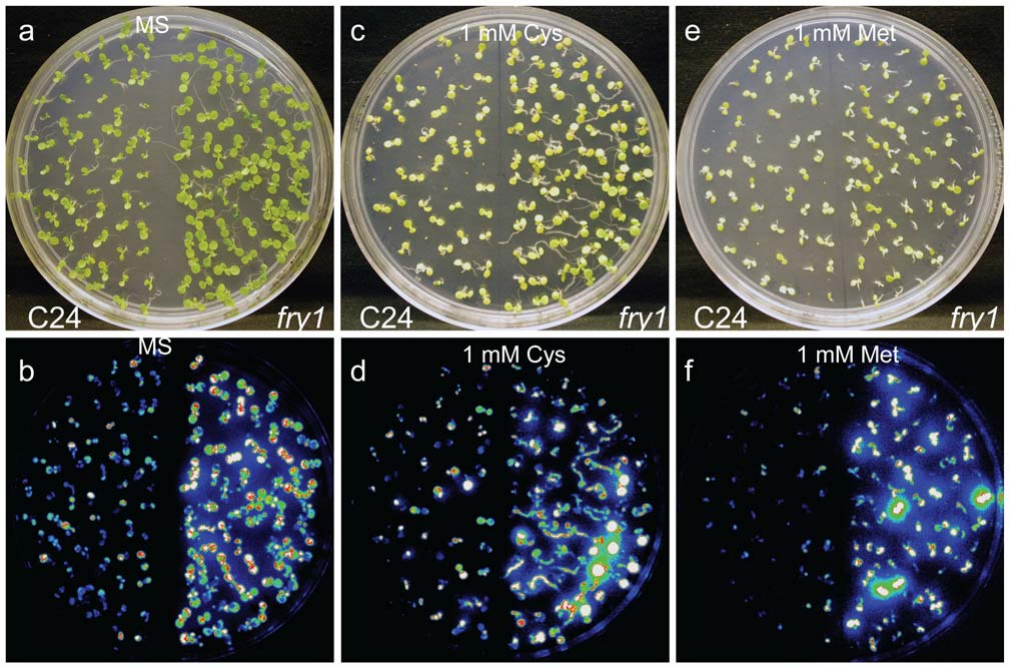
The effect of sulfur-containing amino acids on the induction of the *RD29A-LUC* gene. (a–f) White light (a, c, and e) or luminescence (b, d, f) images of seedlings growing on MS (a and b) or MS supplemented with 1 mM cysteine (Cys; c and d) or 1 mM methionine (Met, e and f) for two weeks. The seedlings were treated at 0°C for 2 days to induce the expression of the *RD29A-LUC* reporter gene before taking the images.

PAP is a byproduct of sulfotransferation reactions from PAPS by PAPS-dependent sulfotransferases and is a competitive inhibitor of such enzymes [9], [21], [22]. PAPS is also an intermediate metabolite of the sulfate activation pathway in bacteria and yeast [23], [24], [25]. Mutations in FRY1 homologs such as the *Escherichia coli cysQ* gene or the yeast *HAL2/MET22* gene resulted in cysteine or methionine auxotrophic growth [23], [24], [25], indicating that PAPS plays an essential role in sulfur assimilation in bacteria and yeast. Moreover, the lithium and sodium tolerance of yeast cells can be improved by methionine supplementation [24], [26], suggesting that sulfate assimilation may be a target of salt toxicity in yeast. Since *fry1* mutants accumulate much higher levels of PAP (Figure 2a), we examined whether the hyperinduction of the reporter gene in *fry1* mutant is caused by PAP inhibition on sulfur assimilation. Wild-type and *fry1* mutant seeds were planted on MS media with or without 1 mM cysteine or methionine, and two-week-old seedlings were examined for the expression of the *RD29A-LUC* reporter gene. It was found that neither cysteine nor methionine was able to restore the reporter gene activity in *fry1* to that of the wild type (Figures 3a to 3f).

### PAP accumulation in mutants of *FRY1* homologs

**Figure 4:**
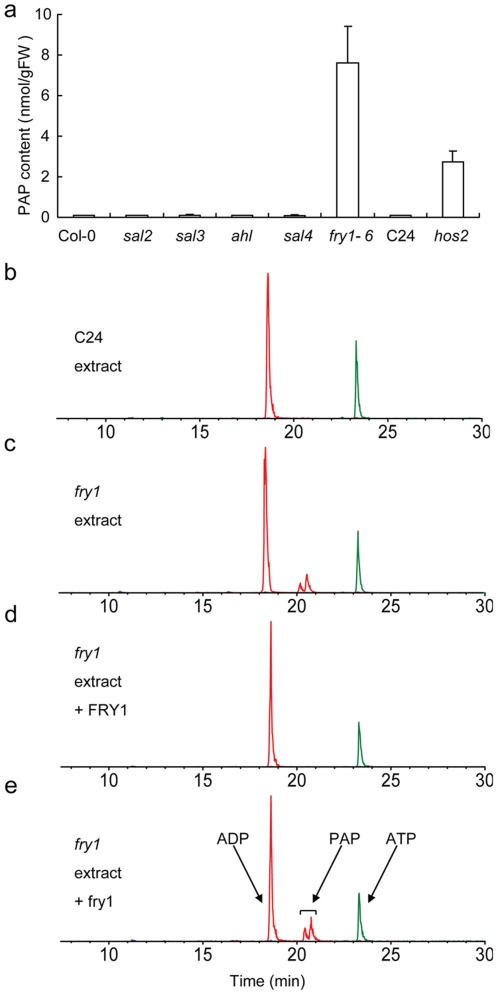
PAP content in wild-type and T-DNA insertion mutants of *FRY1* homologs, and the cleavage of PAP in *fry1* mutant extracts by FRY1 recombinant proteins. (a) PAP content in two-week-old *sal2*, *sal3*, *sal4*, *ahl* or *fry1-6* T-DNA (all in the Col-0 background) or *hos2* mutant (C24 background) seedlings. (b-e) Recombinant wild-type FRY1 but not mutant fry1 protein degraded PAP in *fry1* mutant extracts. Shown are the elution peaks of ADP, ATP, and PAP. Note that the two peaks around 21 min (elution time) both corresponded to PAP (see Materials and Methods for details).

In the Arabidopsis genome, there are four *FRY1* homologs with more than 40% amino acid sequence identity to FRY1, named *SAL2*, *SAL3*, *SAL4*, and *AHL* [7], [8], [10], [11], [20]. Besides FRY1, the *in vitro* nucleotidase activities of SAL2 and AHL have been confirmed with recombinant proteins [7], [8], [20]. All of these genes are transcribed in most of the organs, with *FRY1* and *AHL* having much higher transcript levels than the rest of the family members (https://www.genevestigator.ethz.ch/gv/index.jsp, Figure S1 and [20]). The accumulation of PAP in *fry1* mutant prompted us to examine whether *FRY1* homologs play any role in PAP degradation. Homologous T-DNA insertional mutants of *SAL2*, *SAL3*, *SAL4*, and *AHL* were isolated from the SALK T-DNA collection. Whereas the expression levels of the other lowly expressed members were either too low to be clearly detected or not affected by the particular T-DNA insertions, the respective mutants for the highly expressed members *FRY1* and *AHL* are true knockouts (Figure S2)[11]. PAP content in these seedlings was analyzed. Surprisingly, no significant PAP accumulation was detected in any of these *FRY1* homolog mutants. The PAP contents in these mutants are essentially the same as those in wild-type Col-0 (Figure 4a). In contrast, in *fry1-6*, a *FRY1* T-DNA knockout mutant in the Col-0 background, or in *hos2*, a FRY1 missense mutant in the C24 background [10], the amount of PAP accumulated is either comparable to that in *fry1* or significantly higher than in the wild type (Figures 4a and 2a), indicating that PAP accumulation is not allele- or ecotype-specific.

To confirm the activity of wild type FRY1 and mutant fry1 proteins against PAP, we also carried out an *in vitro* enzymatic activity assay by incubating *E. coli* expressed FRY1 or fry1 protein with nucleotide crude extracts from *fry1* mutant plants. Consistent with *in vitro* assays showing that FRY1/SAL1 possesses strong activity on purified PAP [7], [8], [10] and that the fry1 mutant protein is a loss-of-function protein [7], the PAP peak in the *fry1* nucleotide extract disappeared when incubated with the recombinant wild-type FRY1 protein but not with the fry1 mutant protein (Figures 4b to 4e).

### Subcellular localization of the FRY1 protein

**Figure 5:**
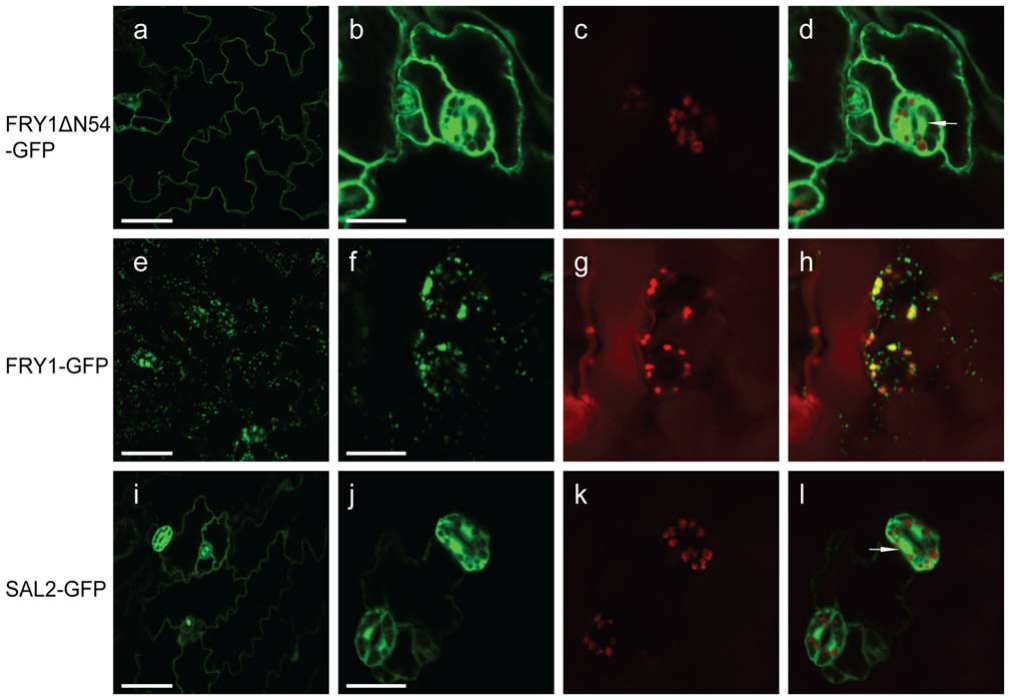
The subcellular localization of the FRY1 (with or without the putative N-terminal signal peptide) and SAL2-GFP fusion proteins. Shown are fluorescence images of the FRY1ΔN54-GFP fusion protein in leaf epidermal cells (a) and guard cells (b to d), the FRY1-GFP fusion protein in leaf epidermal cells (e) and guard cells (f to h), and the SAL2-GFP fusion protein in leaf epidermal cells (i) and guard cells (j to l). Green fluorescence from FRY1ΔN54-GFP (a, b), FRY1-GFP (e, f), and SAL2-GFP (i, j) and fluorescence of chlorophyll (c, g, k) were monitored separately using a confocal laser scanning microscope. (d), (h), and (l) are the merged images of (b) and (c), (f) and (g), and (j) and (k), respectively. Bars = 50 µm in (a), (e) and (i), and 15 µm in (b), (f), and (j). Arrow indicates nucleus in (d) and (l).

When we first characterized the FRY1 protein [7], we checked its subcellular localization (unpublished), yet the interpretation of the data was complicated due to the unique structure of the predicted FRY1 protein. The FRY1 protein has a stretch of 54 aa at its N-terminus (which was not reported in previous studies) that is not present in any other FRY1 family members. This N-terminal stretch is predicated to be a signal peptide for chloroplast targeting [27] and its inclusion [7] or exclusion [8] did not affect the enzymatic activity of the recombinant protein *in vitro*. Interestingly, stromal proteomic analysis detected the presence of the FRY1 protein in the chloroplast [28]. However, transient expression of FRY1-GFP in onion epidermal cell showed that FRY1 was localized either in the cytosol and nuclei [12] or chloroplasts [29]. Here, we generated stable transgenic Arabidopsis plants overexpressing *FRY1* either with or without this putative N-terminal signal peptide with GFP fused in frame at the C-terminus. Multiple independent transgenic plants were examined and similar results were observed. FRY1 without the N-terminal 54 aa (35S-FRY1ΔN54-GFP) was found to be localized to the cytosol and nuclei (Figures 5a to 5d and Figure S3), similar to what was reported in a transient study [12]. However, FRY1 with the N-terminal 54 aa (35S-FRY1-GFP) was localized mainly to chloroplasts (plastids in the roots), but was also found in some unidentified small organelles (Figures 5e to 5h). Both FRY1ΔN54-GFP and FRY1-GFP are functional, as they both complement the *fry1* mutant (see below). We also examined the FRY1 homolog SAL2 that does not have this N-terminus putative signal peptide. SAL2 was localized in the cytosol and the nucleus (Figures 5i to 5l), very similar to FRY1 without the N-terminal 54 aa. It is likely that FRY1 may exist in multiple organelles.

### Overexpression of *FRY1* or its Arabidopsis or yeast homolog rescues *fry1* mutant phenotypes

**Figure 6:**
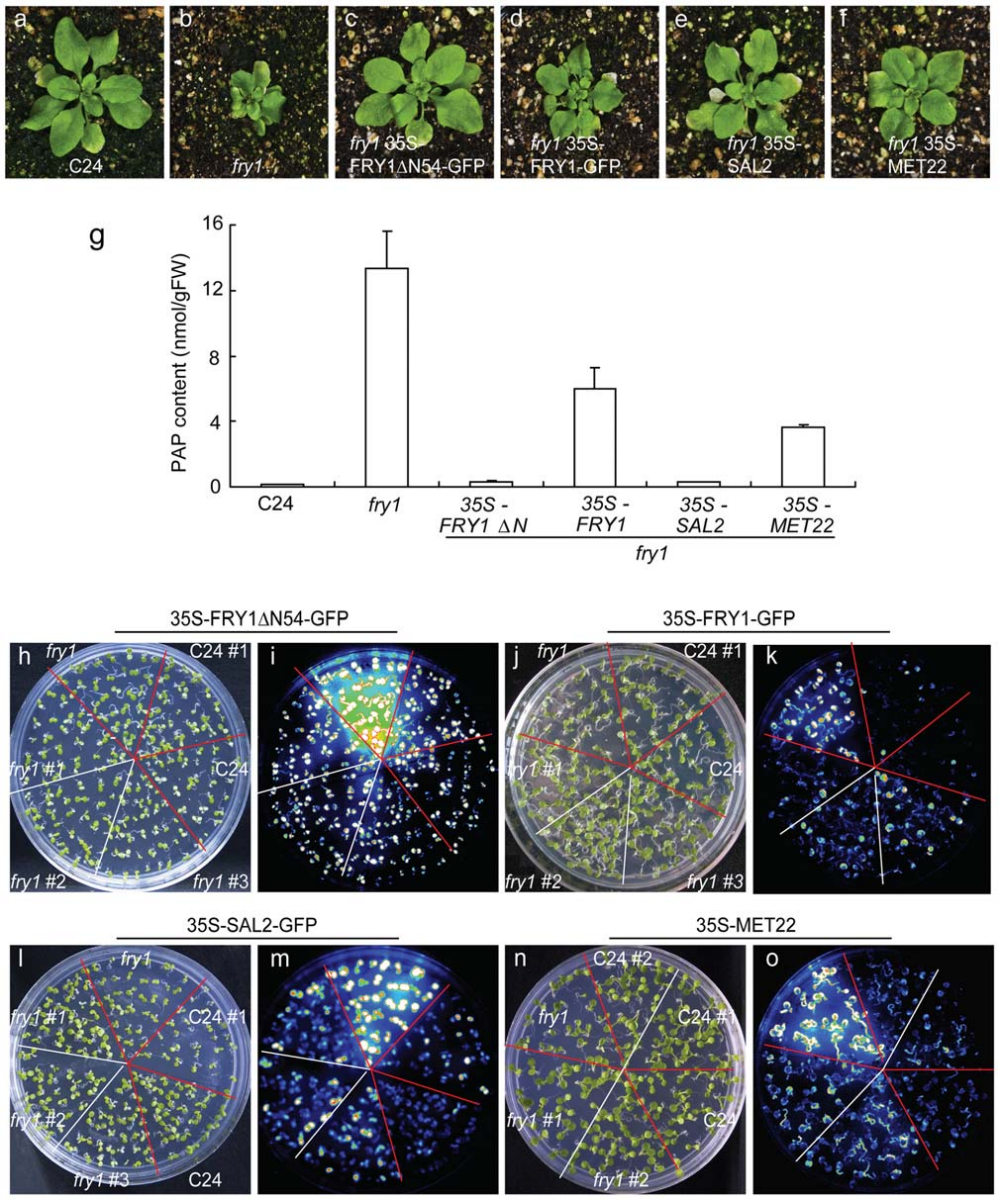
Overexpression of FRY1, SAL2, or MET22 can rescue the morphology, PAP accumulation and *RD29A-LUC* reporter gene expression defects of the *fry1* mutant. (a–f) Morphology of two-week-old, soil-grown seedlings. (g) PAP content in two-week-old C24, *fry1*, *fry1 35S-FRY1ΔN54-GFP*, *fry1 35S-FRY1-GFP, fry1 35S-SAL2,* and *fry1 35S-MET22* seedlings. Data are means and SE from three biological replicates. (h to o) White light images (h, j, l, and n) or luminescence images (i, k, m, and o) of the wild-type C24, *fry1*, or *fry1* mutant expressing the N-terminus-truncated FRY1 (*FRY1ΔN54*) (h and i), the full-length FRY1 (j and k), SAL2 (l and m), or MET22 (n and o) that were treated with cold (0°C) for 2 days after growth on MS medium plates for two weeks. The number sign (#) denotes individual transgenic lines.

The above study indicates that the FRY1 protein has different subcellular localizations, dictated by its N-terminus (Figure 5). Additionally, FRY1 family members may have distinct substrate preferences. To determine the functional relevance of these differences *in planta*, we investigated whether overexpression of FRY1 - when targeted to different locations - or of FRY1 homologs could rescue the diverse *fry1* mutant phenotypes. The unique round and serrated leaves and more compact stature of *fry1* mutants [7], [30] make them easily identifiable. It was found that the N-terminus truncated version can completely restore *fry1* morphology to that of the wild type, while the non-truncated FRY1 protein can significantly but not completely revert the smaller rosette size of the *fry1* mutant (Figures 6a to 6d). Interestingly, overexpression of the Arabidopsis SAL2 or yeast MET22 (also known as HAL2), homologs of FRY1 that catabolize PAP but not IP_3_, also could rescue *fry1* mutant morphological defects, with MET22 being slightly incomplete in the rescue (Figures 6e and 6f). Consistent with their ability to rescue the morphological defects of *fry1* mutants, all of these transgenic lines in the *fry1* background had PAP levels dramatically lower than in *fry1,* and comparable to that of lines overexpressing FRY1ΔN54 and SAL2 in the wild-type background (Figure 6g). Although PAP levels of the other two lines are higher than in the wild type, they are significantly lower than that in the *fry1* mutant (Figure 6g).

We next examined the *RD29A-LUC* reporter gene induction under cold treatment in these transgenic lines together with the *fry1* mutant and the wild type seedlings that also harbored the same reporter gene. It was found that all these transgenes could suppress the superinduction of the reporter gene, with 35S-FRY1ΔN54 (Figures 6h and 6i) and 35S-SAL2 (Figures 6l and 6m) being more effective than 35S-MET22 (Figures 6n and 6o) and 35S-FRY1 (Figures 6j and 6k) (Figure S4). Considering that MET22 only catabolizes PAP but not IP_3_ [9], the result that MET22 overexpression suppressed superinduction of *RD29A-LUC* in the *fry1* mutant indicates that this stress gene superinduction in *fry1* is probably a result of PAP accumulation.

### Mutations in adenosine 5′-phosphosulfate kinases APK1 and APK2 suppress PAP accumulation and superinduction of reporter gene in *fry1* mutant

**Figure 7:**
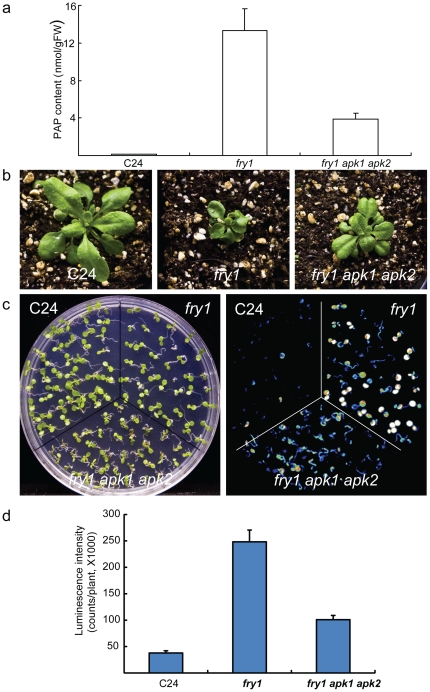
Mutations in both adenosine 5′-phosphosulfate kinases APK1 and APK2 suppress PAP accumulation and the superinduction of the *RD29A-LUC* reporter gene in the *fry1* mutant. (a) PAP content in two-week-old C24, *fry1,* and *fry1apk1 apk2* triple mutant. Data are means and SE from three biological replicates. (b) Morphology of three-week-old soil-grown seedlings of the wild-type C24 (left panel), *fry1* (center panel) and *fry1 apk1 apk2* triple mutant (right panel). The *apk1 apk2* double mutant has similar morphology to the WT (not shown). (c) White light (left panel) or luminescence image (right panel) of the wild-type C24, *fry1*, and *fry1 apk1 apk2* triple mutant seedlings after cold treatment at 0°C for 2 days. Seedlings were grown on MS for ten days at room temperature before the cold treatment. (d) Luminescence intensity of seedlings in (c). Data are means and SE from 25 seedlings.

Adenosine 5′-phosphosulfate kinases (APKs) catalyze the formation of the sulfur donor PAPS [31], [32], [33]. Among members of the APK gene family in Arabidopsis, the transcript levels of *APK1* and *APK2* are much higher than that of *APK3* or *APK4* throughout Arabidopsis development (https://www.genevestigator.ethz.ch/gv/index.jsp, Figure S5). In line with this, *apk1 apk2* double mutant has about a five-fold reduction in glucosinolates, a major class of sulfated secondary metabolites [31], suggesting a greatly reduced biosynthesis of sulfur donor PAPS in the mutant. We hypothesized that with reduced content of the precursor PAPS in the *apk1 apk2* double mutant, this double mutant may also be able to suppress PAP accumulation in the *fry1* mutant. We therefore generated *fry1 apk1 apk2* triple mutant by crossing *fry1* with *apk1* and *apk2*. Indeed, the LC-MS/MS analysis indicated that the PAP content of the triple mutant was reduced over 70 percent compared to the *fry1* mutant (Figure 7a). The morphology of the triple mutant also looked nearly normal and the seedling size was much bigger than the original *fry1* mutant (Figure 7b).

We then checked the reporter gene expression in this triple mutant. It was found that cold induction of the reporter gene was significantly reduced compared to the *fry1* mutant, although it was still higher than that of the wild type (Figures 7c and 7d). These results further support the notion that PAP is critical for the superinduction of stress responsive genes in the *fry1* mutant.

### ABA and stress superinduction of *RD29A-LUC* in *fry1* is dependent on ABH1 but not ABI1

**Figure 8:**
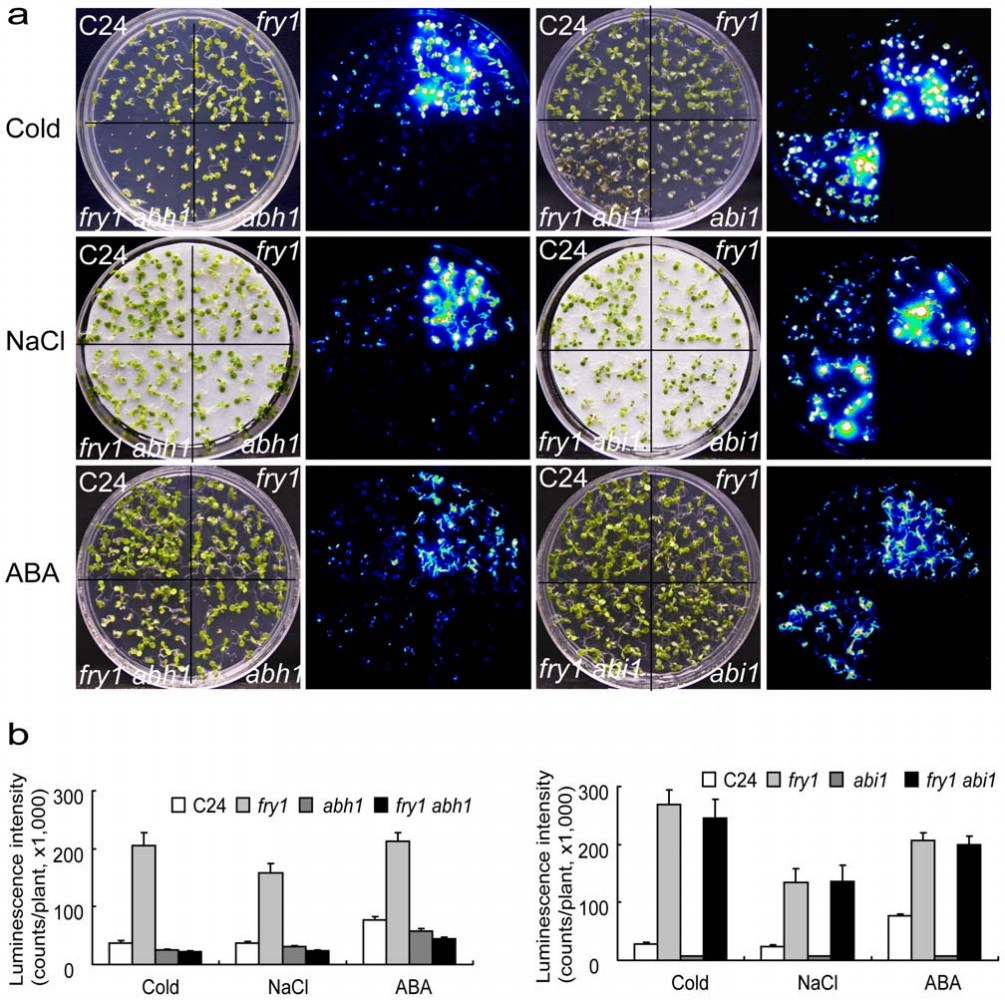
A mutation in ABH1 but not ABI1 can suppress the superinduction of *RD29A-LUC* in *fry1* mutant. (a) White light or luminescence images of C24, *fry1*, *fry1 abh1* or *fry1 abi1* double mutant seedlings after cold (0°C, 2 days), NaCl (300 mM, 3 hr), or ABA (100 µM, 3 hr) treatment. The seedlings were grown on MS media for two weeks before being incubated in the cold, sprayed with ABA or transferred to salt-saturated filter paper for the respective stress treatment. The *abi1-1* single mutant did not contain the *RD29A-LUC* transgene and cannot be used to directly compare with other lines for luminescence expression (the luminescence intensity for *abi1* in (b) reflects background readings). (b) Luminescence intensity of seedlings in (a). Data are means and SE (*n* ≥ 15).

Many stress-signaling pathways that lead to the activation of stress-responsive genes are dependent on ABA signaling [2], [34]. ABI1 is a key component downstream of ABA receptors in the ‘core’ ABA signaling pathway [35], [36], [37], whereas some other regulators such as ABH1 [38], HYL1 [39] and SAD1 [40] appear to mediate other ABA signaling branches related to mRNA metabolism. The relatedness of these different ABA signaling pathways is currently unclear. The *abh1* mutant defective in an mRNA cap binding protein is hypersensitive to ABA in stomata closure [38]. The uncapped mRNAs are proposed to be one of the substrates for ribonuclease XRN4 [41], which likely is inhibited by PAP. We thus hypothesized that FRY1 and ABH1 may act through the same pathway. In our genetic screen, we also isolated an allele of ABH1, *abh1-7*, that exhibited reduced ABA induction of the *RD29A-LUC* expression [40], [42]. To explore the relationship between these different ABA signaling components involved in the regulation of stress responsive genes, we generated the double mutants *fry1 abh1* and *fry1 abi1*. These double mutants along with the single mutants were treated with cold, NaCl, or ABA, and the induction of *RD29A-LUC* was examined. Interestingly, the dominant negative *abi1-1* mutation had no effect on the superinduction of the reporter gene in *fry1* under all conditions tested (Figures 8a and 8b). In sharp contrast, *abh1* almost completely suppressed the superinduction of *RD29A-LUC* in *fry1* after either cold, NaCl, or ABA treatment (Figures 8a and 8b).

### *FRY1* overexpression in Arabidopsis does not confer tolerance to salt stress

**Figure 9:**
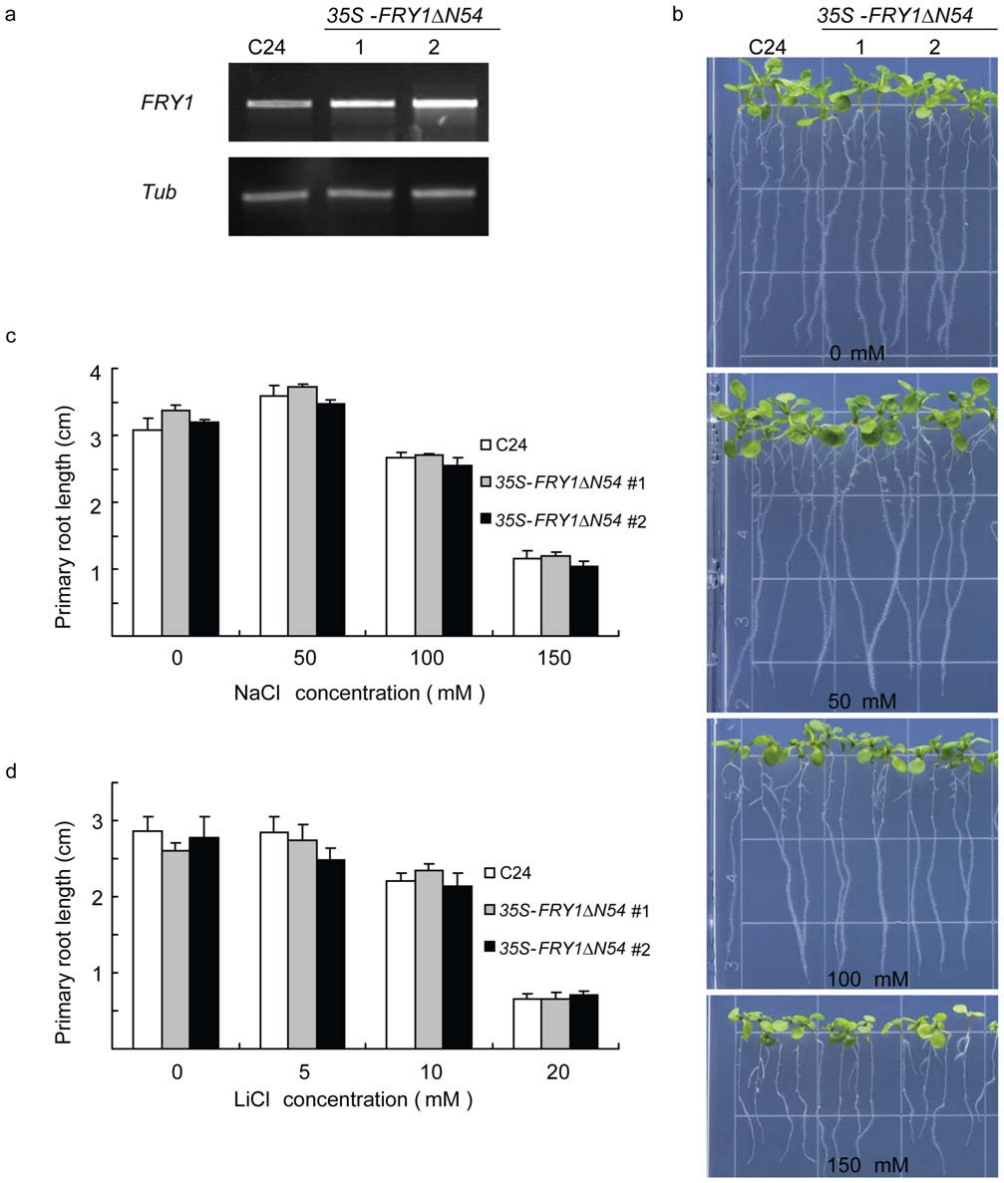
Overexpression of *FRY1* does not confer enhanced salt tolerance. (a) RT-PCR of *FRY1* in two random transgenic lines expressing an N-terminal-truncated FRY1 (FRY1Δ54). (b) Morphology of wild-type C24 and *35S-FRY1Δ54* transgenic lines on MS agar plates with 0, 50, 100, or 150 mM NaCl. Five-day-old seedlings were transferred from MS agar plates to plates containing the indicated concentrations of NaCl, and pictures were taken ten days after the transfer. (c) Primary root length of wild-type C24 and *35S-FRY1* seedlings on MS agar plates containing 0, 50, 100, or 150 mM NaCl. Five-day-old seedlings were transferred from regular MS agar plates to plates containing the indicated concentrations of NaCl, and the primary root length was measured one week after the transfer. Data are means and SE (*n* = 10). (d) Primary root length of wild-type C24 and *35S-FRY1Δ54* seedlings on MS plates containing 0, 5, 10, or 20 mM LiCl. Five-day-old seedlings were transferred from MS plates to MS plates containing the indicated concentrations of LiCl, and the primary root length was measured one week after the transfer. Data are means and SE (*n* = 10).

Although overexpression of either yeast *HAL2* or *FRY1* can confer salt tolerance to yeast [8], [43], it is not clear whether upregulating *FRY1* expression can enhance salt tolerance in plants. We generated transgenic Arabidopsis expressing the CaMV 35S promoter-driven *FRY1* without the N-terminal 54 aa, given that this version of FRY1 is more effective in rescuing both *fry1* morphology and the reporter gene induction (Figures 6c and 6i). Two independent transgenic lines were randomly chosen to test salt tolerance. Both lines had increased levels of the steady-state *FRY1* transcript (Figure 9a). When subjected to NaCl stress, the FRY1-overexpressing seedlings did not exhibit any clear difference in salt tolerance from the wild type, judged either by root elongation or seedling growth (Figures 9b and 9c). These transgenic seedlings were not more resistant to Li^+^ inhibition of primary root growth either (Figure 9d). Since the 35S promoter driven *FRY1*ΔN54 construct could restore PAP level to normal in *fry1* (Figure 6g), it is very likely that the overexpressed FRY1ΔN54 is functional in PAP cleavage. In addition, we generated transgenic Arabidopsis expressing the full-length *FRY1* under the control of the 35S promoter. However, no clear change in tolerance to salt or other stress was observed in the transgenics either (data not shown). Therefore, unlike in yeast, the 3′, 5′-bisphosphate nucleotidase activity of FRY1 may not be a primary target of sodium or lithium toxicity in Arabidopsis.

## Discussion

The nucleotide 3′-phosphoadenosine-5′-phosphate (PAP) is a byproduct of sulfotransferation reactions and sulfur assimilation. In bacteria and yeast, sulfate activation is achieved by the formation of the high-energy sulfur donor PAPS, while its de-sulfated product PAP acts as a strong competitive inhibitor of PAPS-based sulfotransferases [9], [20]. As a result, null mutations in *E. coli cysQ* gene or yeast *HAL2/MET22* gene will lead to the accumulation of PAP and blockage of sulfate assimilation. These mutants exhibit cysteine or methionine auxotrophic growth [23], [24], [25]. The Arabidopsis homolog to these genes is *FRY1*, although the function may not be the same. Since *fry1* mutants are viable on MS medium where sulfate is the sole source of sulfur, FRY1 is most probably not involved in sulfur assimilation in plants. To rule out the possibility that sulfur deficiency causes the superinduction of stress-responsive genes in *fry1* mutant, we supplemented the growth media with either cysteine or methionine. Yet, these reduced sulfur donors could not repress the superinduction of the *RD29A-LUC* reporter gene (Figures 3d and 3f). The hypersensitive induction of the reporter gene in *fry1* mutants was thus not caused by inhibited sulfur assimilation. Our data also suggest that high levels of PAP may not interfere with sulfur assimilation in Arabidopsis. This is consistent with the notion that the pathway of sulfate assimilation in higher plants differs from that of bacteria and yeast through bypassing the PAPS but using APS reductase to reduce sulfate [44], [45], [46].

In addition to inhibiting sulfate assimilation in yeast, the accumulation of PAP beyond a threshold level will be toxic to other cellular processes [26], [47]. Enzymes that can hydrolyze PAP thus become critical under conditions where PAP may accumulate. When grown under high salt conditions, the yeast 3′,5′-bisphosphate nucleotidase HAL2 becomes inhibited and thus is an in vivo target of salt toxicity [43]. Since the Arabidopsis SAL1/FRY1 was able to restore salt tolerance of the yeast *hal2* salt-sensitive mutant, SAL1/FRY1 was considered as a potential target of salt toxicity in plants [8]. To investigate whether higher plants have similar salt-sensitive targets, we first needed to determine whether salt (and other stresses) could lead to an increased accumulation of PAP. To this end, we developed an LC-MS/MS method that allows us to quantitatively measure PAP levels in plants. Using this method, we found that although both NaCl and LiCl can greatly inhibit FRY1 activity *in vitro* [8], [10], neither led to increased accumulation of PAP in plants (Figures 2a, 2d, and 2f). Other stimuli, such as cold or ABA, did not increase PAP levels either (Figures 2g and 2h). These data indicated that these stress treatments had little effect on FRY1's catalytic activity on PAP *in vivo.* Contrary to the very low levels of PAP in wild type, however, *fry1* mutants did have significantly higher levels of PAP (Figures 2 and 4a). Nonetheless, despite the extremely high level of PAP, *fry1* mutants only exhibited a moderate sensitivity to salt stress at the seedling stage [10], much less than the sensitivity seen with the Arabidopsis *sos* mutants [48]. Even this limited sensitivity of *fry1* mutant plants to salt at the seedling stage could no longer be observed when the plants reached the adult stage (data not shown). Transgenic plants overexpressing FRY1 also did not show enhanced tolerance to either NaCl or LiCl (Figure 9). In fact, adult *fry1* mutant plants appeared to be more tolerant to drought stress [49], [50] and tended to have less leaf-bleaching under salt stress (data not shown). Thus, unlike in yeast, inhibition of the FRY1 activity on PAP catabolism does not appear to be the cause of salt toxicity in higher plants.

The Arabidopsis FRY1 family contains several other members that also exhibit PAP nucleotidase activity *in vitro*. Surprisingly, high accumulation of PAP was only detected in *fry1* mutants and not in other examined mutants in the same gene family (Figures 2 and 4a), suggesting that FRY1 is the major, if not the only, enzyme that catalyzes the degradation of PAP *in planta*. This is interesting since *FRY1* and *AHL* have comparable transcript levels, although the transcript levels of *SAL2*, *SAL3*, and *SAL4* are much lower than that of *FRY1* (https://www.genevestigator.ethz.ch/gv/index.jsp and [20]). This may have to do with the fact that FRY1 has much higher affinity and activity than AHL toward PAP. It was shown that the affinity to PAP of FRY1 is about sixteen- to eighty-fold that of AHL, while the activity of FRY1 is about six-fold that of AHL [20]. Despite the high level of PAP in *fry1*, there was no apparent change in the levels of AMP, ADP, or ATP in the mutants (Figure 2b), indicating that accumulation of PAP does not seem to interfere with the metabolism of other adenine nucleotides.

It is interesting to note that both the N-terminally truncated FRY1 (FRY1ΔN54-GFP; cytosol and nucleus localized) and the full-length FRY1-GFP (mainly chloroplast localized) can complement (or partially complement) *fry1* mutant in morphology, PAP accumulation, and reporter gene expression (Figures 5 and 6). Although the exact subcellular localization of the endogenous FRY1 protein is currently unknown, these results suggest that PAP can be transported among cellular compartments. In addition to FRY1, other enzymes involved in sulfate assimilation are also localized in multiple cellular locations. For instance, ATP sulfurylase activities for the production of APS were detected in both chloroplasts and the cytosol [51], [52]. Similarly, Arabidopsis APS kinases for PAPS biosynthesis are found in either chloroplasts or the cytosol [31]. Therefore, the PAP precursor PAPS can be synthesized in both chloroplasts and the cytosol, although the sulfotransferases that use PAPS as a sulfur donor are mostly localized in the cytosol [45], [53]. Presumably, chloroplast-synthesized PAPS can be readily transported into the cytosol for sulfation. Several PAPS transporters have been identified in *Drosophila* and mammals [54], [55], [56], [57], [58], although plant PAP or PAPS transporters have not been reported.

As a bifunctional enzyme, FRY1/SAL1 also exhibits inositol polyphosphate 1-phosphatase activity, which is presumed to play important roles in phosphoinositide signaling [7], [8]. Indeed, a significant increase in the level of inositol 1,4,5-trisphosphate was detected in *fry1* mutants following cold treatments [7]. It was proposed that the superinduction of the *RD29A*-*LUC* reporter gene as well as endogenous stress-responsive genes in this mutant may be due to its inability to desensitize IP_3_ signaling following stress or ABA treatment [7]. However, the contribution of either the inositol polyphosphatase or the nucleotide bisphosphatase activity to stress gene regulation was not completely resolved in previous studies. Recently FRY1 was also identified as a suppressor of RNA silencing [11]. It was proposed that FRY1 mediates RNA silencing through exoribonucleases XRN2, XRN3, and XRN4 [11], [59], since PAP is a strong inhibitor of these RNA-metabolizing enzymes [26], [47]. Several recent studies suggested that the inhibition of *XRN* may be linked to some of the *fry1* phenotypes, such as reduced lateral root growth [14] and light sensitivity [12], [13]. We thus investigated whether *XRN* inhibition is also responsible for the superinduction of stress-induced *RD29A-LUC* expression. When *xrn2*, *xrn3*, or *xrn4* mutation was introduced into the wild type C24 (containing the *RD29A-LUC* reporter) background, we did not observe the superinduction of the reporter gene upon treatment with stress or ABA (data not shown). This finding suggests that either PAP inhibition of XRNs is not responsible for the superinduction of the reporter gene or that the XRNs function redundantly.

To determine whether the superinduction of stress-responsive *RD29A-LUC* in *fry1* is caused by its inability to catalyze PAP degradation, we employed two approaches to prevent PAP accumulation in *fry1*. Firstly, we overexpressed the yeast FRY1 homolog MET22, which has a strong *in vitro* catalytic activity on PAP but virtually no *in vitro* activity on IP_3_ [9]. Secondly, we generated a triple mutant of *fry1* with *apk1* and *apk2*, two adenosine 5′-phosphosulfate kinase mutants defective in the formation of the PAP precursor PAPS [31]. With either approach, we could significantly reduce the PAP level in the *fry1* mutant background (Figures 6g and 7a). With the greatly reduced PAP level in these *fry1* mutant plants, ABA or stress induction of *RD29A-LUC* was greatly reduced or diminished to close to that in the wild type (Figures 6o, 7c, and S4). These results demonstrate that PAP is likely responsible for the superinduction of *RD29A-LUC* in *fry1* mutants. Furthermore, genetic analysis indicated that there is an interaction between FRY1 and mRNA metabolism, since mutation in the mRNA cap-binding protein ABH1 could suppress the superinduction of *RD29A-LUC* in the *fry1* mutant as well (Figure 8). In contrast, the core ABA signaling component ABI1 seems to not be involved in this process (Figure 8), implying that the pathways leading to stress or ABA superinduction of *RD29A-LUC* in *fry1* may not be through the recently identified core ABA signal pathway [60], [61], [62].

## Materials and Methods

### Plant materials, growth conditions, and stress treatments

The *Arabidopsis thaliana* ethyl methanesulfonate (EMS)-mutated lines *fry1-1* (referred as *fry1*) and *hos2* in the C24 background (with the transgene *RD29A-LUC*, referred as the ‘wild type’ in the text) were reported previously [7], [10], [63]. The homozygous T-DNA insertional lines SALK_020882 (*fry1-6*), SALK_055685 (*ahl*), SALK_085187 (*sal4*), SALK_101315 (*sal2*), SALK_065642C (*sal3*), SALK_053427 (*apk1*), and SALK_077590 (*apk2*) in the Col-0 background were isolated from the SALK T-DNA library by using PCR-based genotyping. The *abh1-7* mutant allele was isolated in our genetic screen [40], [42]. Seeds were surface-sterilized with bleach plus 0.01% triton and planted onto a half-strength Murashige and Skoog (MS) medium supplemented with 3% sucrose and 0.6% agar (Sigma, St Louis, MO). After two days of cold treatment, plates were incubated at 22°C under constant white light for seed germination and seedling growth.

Stress treatments for PAP analysis and luminescence imaging were carried out as follows. For ABA, cold, and short-term salt treatments, two-week-old seedlings grown in a MS medium in agar Petri dishes were either treated for a designated time in the cold (0°C), sprayed with 100 µM ABA, or transferred to filter papers saturated with 300 mM NaCl solution. For longer-term salt treatment, wild-type C24 and *fry1* seeds were directly planted on MS or MS supplemented with 50 or 100 mM NaCl, or with 5 or 10 mM LiCl and seedlings were allowed to grow for two-weeks. Samples were then harvested and PAP was extracted and measured as described below. Chlorophyll *a*, *b*, and carotenoid contents were measured as described [64]. Luminescence imaging was performed as described [4].

For salt tolerance test of FRY1 overexpression lines, five-day-old seedlings were transferred to MS plates with or without 50, 100, or 150 mM NaCl and plates were incubated vertically and seedlings were photographed using a digital camera ten days after the transfer.

### PAP Analysis

PAP was quantified by using a 4000 QTRAP LC-MS/MS (Applied Biosystems, Foster City, CA). Liquid nitrogen-frozen *Arabidopsis* fresh tissues (in amounts as low as 10 mg) were ground into a fine powder with TissueLyser II (Qiagen, Valencia, CA) and extracted with −20°C cold chloroform, methanol and acetonitrile (2∶1∶1 v/v/v) with 0.4% formic acid in 1.5 mL Eppendorf tubes. Ice-cold water was then added for phase partitioning. The polar phase, which contains PAP, was collected and the chloroform phase was back-extracted once. Combined polar extracts were lyophilized and stored at −80°C until further analysis.

For LC-MS/MS quantification of PAP, dried extracts were dissolved in 100 µL ice-cold water with 3.5 mM ammonium acetate, and 10 µL was injected for analysis. PAP separation was achieved by ion pair chromatography with an Onyx monolithic C18 column (100×3.0 mm with guard cartridge, Phenomenex, Torrance, CA) using Solvent A: 10 mM n-hexylamine in 5% acetonitrile, pH adjusted to 6.5±0.2 with acetic acid and Solvent B: 10 mM ammonium acetic acid in 90% methanol at a flow rate of 1.0 ml/min. The LC gradient was from 0% B to 100% B over 9 minutes, followed by a hold time of 2 minutes at 100% B, and a re-equilibration at 0% B for 3 minutes. The LC-MS/MS system was composed of a LEAP autosampler, Shimadzu binary solvent delivery system, UV detector and 4000 QTRAP mass spectrometer equipped with TurboIonSpray ion source. PAP was eluted after ADP and before ATP with baseline resolution. Ion source parameters were: CUR 25, TEM 550, GS1 50, GS2 55, and CAD High. Since no ion suppression was found, an external calibration curve using PAP standards was employed for quantification of endogenous PAP levels in tissue extracts.

### In vitro enzymatic assay

For treatment of crude nucleotide extracts with *E. coli*-expressed recombinant enzymes, wild-type FRY1 and fry1 mutant protein were expressed and purified as described [7], and enzyme assay was performed as described for 1 hr at 37°C [9]. The enzyme-treated extract was then analyzed with a slight revision to the above-described LC-MS/MS method. This minor modification was necessary for two reasons. Firstly, the above-described method is a quick LC method devoted to PAP measurement. However, several additional nucleotides, including NADP, NAD, NADPH, and NADP, eluted before ADP or AMP, where ion suppression is more prone to happen. Secondly, adding more MRM transitions to existing MRM tables increased the total cycle time (a sum of dwell time and settling time for each MRM channel), however, the narrow LC peak does not permit longer total cycle time. Therefore, in order to better resolve nucleotides other than ADP and PAP, two tandem Onyx monolithic C18 columns (4.6 ID ×100 mm, Phenomenex, CA) were used, and the LC eluting program was changed accordingly. The new LC program used Solvent A: 10 mM n-hexylamine in 5% acetonitrile, pH adjusted to 6.5±0.2 with acetic acid and Solvent B: 10 mM ammonium acetic acid in 90% methanol at a flow rate of 1.0 ml/min. The LC gradient was from 0% B to 40% B over 20 min, followed by a gradient from 40% B to 100% B over 2 min then a hold time of 6 min at 100% B, and a re-equilibration at 0% B for 6 min. The autosampler injection volume was set to 50 µL. Mass spectrometric data were recorded from 1.2 to 30 min during the LC run. For MRM detection of nucleotides and related compounds, MRM transitions were added for corresponding compounds optimized based on in-house collection of standards, and for phosphate groups with different declustering potentials which would capture any potential significant changes of metabolites containing phosphate group. However, only PAP was detected as the major signal with corresponding changes.

Under these conditions, two peaks with the same MRM transition were found to be associated with PAP, and both have similar MRM transitions and ratios. The reason is not completely clear, either due to the endogenous unknown enzymatic reactions or due to the analytical artifacts (such as unknown isomerization reactions in solution). It should be noted that PAP could have two isomers, i.e., 3′-phosphoadenosine -5′-phosphate or 2′-phosphoadensine-5′-phosphate, and that both isomers are substrates of FRY1 [8]. Therefore, we summed the two peaks together to represent the total PAP levels.

### Plant transformation

The following primer pairs 5′-CACCATGATGTCTATAAATTGTTTTCG and 5′-GAGAGCTGAAGCTTTCTCTT or 5′-CACCATGGCTTACGAGAAAGAGCT and 5′-GAGAGCTGAAGCTTTCTCTT were used to amplify *FRY1* cDNAs with or without the N-terminal 54 aa signal peptide, respectively. *SAL2* cDNA was amplified with the primers 5′-ATGTCTTATGAGAAGGAGCT and 5′-GAAATAAAGATTCTCTTCTTCTATG. Yeast *MET22* genomic DNA was amplified with the primers 5′-CACCATGGCATTGGAAAGAGAATTATT and 5′-GGCGTTTCTTGACTGAATGA. The DNA fragments were ligated into the pENTR-D-TOPO vector (Invitrogen, Carlsbad, CA). After sequence confirmation, they were cloned into pMDC Gateway vector pMDC83 in frame with GFP coding sequence to generate 35S-FRY1-GFP, 35S-FRY1ΔN54-GFP, and 35S-SAL2-GFP or into pMDC32 to generate 35S-MET22 through LR clonase recombination. *Agrobacterium tumefaciens* GV3101 were transformed by electroporation with the constructs. Four-week-old Arabidopsis were then vacuum-infiltrated with the transformed *Agrobacterium* strains and transformants were screened using hygromycin. GFP fluorescent signal was visualized as described [65].

### RNA analysis

For expression analysis of CaMV 35S promoter driven *FRY1* overexpression lines, total RNA was extracted from seedlings grown under a long-day photoperiod by using TRIZOL Reagent according to the manufacturer's manual (Invitrogen, Carlsbad, CA). For RT-PCR analysis, cDNA was synthesized using oligo dT primer and moloney murine leukemia virus (MMLV) reverse transcriptase (New England Biolabs, Boston, MA), and PCR was performed with the primers 5′-ATGGCTTACGAGAAAGAGCT and 5′-GAGGCATCCAATTTCGTCTGA. The primer pair for tubulin amplification was 5′- CGTGGATCACAGCAATACAGAGCC and 5′- CCTCCTGCACTTCCACTTCGTCTTC.

## Supporting Information

Figure S1 The transcript levels of *FRY1* family members at different developmental stages. Data were extracted from the public microarray database (https://www.genevestigator.ethz.ch).
(TIF)

Figure S2 RT-PCR analysis of T-DNA insertion lines of *FRY1* homologs. cDNA from T-DNA lines was amplified with primers specific to coding regions of FRY1 homologs; Col-0 is included as a positive control.
(TIF)

Figure S3 The subcellular localization of the FRY1ΔN54-GFP fusion protein in root epidermal cells. Green fluorescence from FRY1ΔN54-GFP (a) and DAPI fluorescence of stained nuclei (b) were monitored separately using an epifluorescence microscope (Nikon Eclipse E800). (c) is the merged images of (a) and (b). Bar = 50 µm. Arrow indicates nucleus in (b).
(TIF)

Figure S4 Relative luminescence intensity of seedlings in Figure 6. Data are means and SE from more than 25 seedlings.
(TIF)

Figure S5 The transcript levels of *APK* family members at different developmental stages. Data were extracted from the public microarray database (https://www.genevestigator.ethz.ch).
(TIF)

## References

[1] Yamaguchi-Shinozaki K, Shinozaki K (2006). Transcriptional regulatory networks in cellular responses and tolerance to dehydration and cold stresses.. Annu Rev Plant Biol.

[2] Zhu JK (2002). Salt and drought stress signal transduction in plants.. Annu Rev Plant Biol.

[3] Thomashow MF (1999). Plant cold acclimation: freezing tolerance genes and regulatory mechanisms.. Annu Rev Plant Physiol Plant Mol Biol.

[4] Ishitani M, Xiong L, Stevenson B, Zhu JK (1997). Genetic analysis of osmotic and cold stress signal transduction in Arabidopsis: interactions and convergence of abscisic acid-dependent and abscisic acid-independent pathways.. Plant Cell.

[5] Brown E, Malakar S, Krebs JE (2007). How many remodelers does it take to make a brain? Diverse and cooperative roles of ATP-dependent chromatin-remodeling complexes in development.. Biochem Cell Biol.

[6] Koiwa H, Bressan RA, Hasegawa PM (2006). Identification of plant stress-responsive determinants in Arabidopsis by large-scale forward genetic screens.. J Exp Bot.

[7] Xiong L, Lee B, Ishitani M, Lee H, Zhang C (2001). *FIERY1* encoding an inositol polyphosphate 1-phosphatase is a negative regulator of abscisic acid and stress signaling in *Arabidopsis*.. Genes Dev.

[8] Quintero FJ, Garciadeblas B, Rodriguez-Navarro A (1996). The *SAL1* gene of Arabidopsis, encoding an enzyme with 3′(2′),5′-bisphosphate nucleotidase and inositol polyphosphate 1-phosphatase activities, increases salt tolerance in yeast.. Plant Cell.

[9] Murguia JR, Belles JM, Serrano R (1995). A salt-sensitive 3′(2′),5′-bisphosphate nucleotidase involved in sulfate activation.. Science.

[10] Xiong L, Lee H, Huang R, Zhu JK (2004). A single amino acid substitution in the Arabidopsis FIERY1/HOS2 protein confers cold signaling specificity and lithium tolerance.. Plant J.

[11] Gy I, Gasciolli V, Lauressergues D, Morel J-B, Gombert J (2007). Arabidopsis FIERY1, XRN2, and XRN3 are endogenous RNA silencing suppressors.. Plant Cell.

[12] Kim BH, von Arnim AG (2008). FIERY1 regulates light-mediated repression of cell elongation and flowering time via its 3′(2′),5′-bisphosphate nucleotidase activity.. Plant J.

[13] Chen H, Xiong L (2011). Genetic interaction of two abscisic acid signaling regulators, HY5 and FIERY1, in mediating lateral root formation.. Plant Signal Behav.

[14] Chen H, Xiong L (2010). The bifunctional abiotic stress signalling regulator and endogenous RNA silencing suppressor FIERY1 is required for lateral root formation.. Plant Cell Environ.

[15] Li E (2002). Chromatin modification and epigenetic reprogramming in mammalian development.. Nat Rev Genet.

[16] Rabinowitz JD, Kimball E (2007). Acidic acetonitrile for cellular metabolome extraction from Escherichia coli.. Anal Chem.

[17] Fiehn O, Kopka J, Dormann P, Altmann T, Trethewey RN (2000). Metabolite profiling for plant functional genomics.. Nat Biotechnol.

[18] Gibon Y, Vigeolas H, Tiessen A, Geigenberger P, Stitt M (2002). Sensitive and high throughput metabolite assays for inorganic pyrophosphate, ADPGlc, nucleotide phosphates, and glycolytic intermediates based on a novel enzymic cycling system.. The Plant journal: for cell and molecular biology.

[19] Stitt M (1986). Limitation of photosynthesis by carbon cetabolism: I. Evidence for excess electron transport capacity in leaves carrying out photosynthesis in saturating light and CO(2).. Plant Physiol.

[20] Gil-Mascarell R, Lopez-Coronado JM, Belles JM, Serrano R, Rodriguez PL (1999). The *Arabidopsis HAL2*-like gene family includes a novel sodium-sensitive phosphatase.. Plant J.

[21] Schmidt A, Jager K (1992). Open questions about sulfur metabolism in plants.. Annu Rev Plant Physiol Plant Mol Biol.

[22] Leustek T, Martin MN, Bick JA, Davies JP (2000). Pathways and regulation of sulfur metabolism revealed through molecular and genetic studies.. Annu Rev Plant Physiol Plant Mol Biol.

[23] Neuwald AF, Krishnan BR, Brikun I, Kulakauskas S, Suziedelis K (1992). *cysQ*, a gene needed for cysteine synthesis in *Escherichia coli* K-12 only during aerobic growth.. J Bacteriol.

[24] Glaser HU, Thomas D, Gaxiola R, Montrichard F, Surdin-Kerjan Y (1993). Salt tolerance and methionine biosynthesis in *Saccharomyces cerevisiae* involve a putative phosphatase gene.. EMBO J.

[25] Thomas D, Barbey R, Henry D, Surdin-Kerjan Y (1992). Physiological analysis of mutants of *Saccharomyces cerevisiae* impaired in sulphate assimilation.. J Gen Microbiol.

[26] Dichtl B, Stevens A, Tollervey D (1997). Lithium toxicity in yeast is due to the inhibition of RNA processing enzymes.. EMBO J.

[27] Emanuelsson O, Brunak S, von Heijne G, Nielsen H (2007). Locating proteins in the cell using TargetP, SignalP and related tools.. Nature Protocols.

[28] Peltier JB, Cai Y, Sun Q, Zabrouskov V, Giacomelli L (2006). The oligomeric stromal proteome of *Arabidopsis thaliana* chloroplasts.. Mol Cell Proteomics.

[29] Rodriguez VM, Chetelat A, Majcherczyk P, Farmer EE (2010). Chloroplastic phosphoadenosine phosphosulfate (PAPS) metabolism regulates basal levels of the prohormone jasmonic acid in Arabidopsis leaves.. Plant Physiol.

[30] Robles P, Fleury D, Candela H, Cnops G, Alonso-Peral MM (2010). The *RON1/FRY1/SAL1* gene is required for leaf morphogenesis and venation patterning in Arabidopsis.. Plant Physiol.

[31] Mugford SG, Yoshimoto N, Reichelt M, Wirtz M, Hill L (2009). Disruption of adenosine-5′-phosphosulfate kinase in Arabidopsis reduces levels of sulfated secondary metabolites.. Plant Cell.

[32] Lillig CH, Schiffmann S, Berndt C, Berken A, Tischka R (2001). Molecular and catalytic properties of *Arabidopsis thaliana* adenylyl sulfate (APS)-kinase.. Arch Biochem Biophys.

[33] Lee S, Leustek T (1998). APS kinase from *Arabidopsis thaliana*: genomic organization, expression, and kinetic analysis of the recombinant enzyme.. Biochem Biophys Res Commun.

[34] Finkelstein RR, Gampala SS, Rock CD (2002). Abscisic acid signaling in seeds and seedlings..

[35] Park SY, Fung P, Nishimura N, Jensen DR, Fujii H (2009). Abscisic acid inhibits type 2C protein phosphatases via the PYR/PYL family of START proteins.. Science.

[36] Ma Y, Szostkiewicz I, Korte A, Moes D, Yang Y (2009). Regulators of PP2C phosphatase activity function as abscisic acid sensors.. Science.

[37] Nishimura N, Sarkeshik A, Nito K, Park SY, Wang A (2010). PYR/PYL/RCAR family members are major in-vivo ABI1 protein phosphatase 2C-interacting proteins in Arabidopsis.. Plant J.

[38] Hugouvieux V, Kwak JM, Schroeder JI (2001). An mRNA cap binding protein, ABH1, modulates early abscisic acid signal transduction in Arabidopsis.. Cell.

[39] Lu C, Fedoroff N (2000). A mutation in the Arabidopsis *HYL1* gene encoding a dsRNA binding protein affects responses to abscisic acid, auxin, and cytokinin.. Plant Cell.

[40] Xiong L, Gong Z, Rock CD, Subramanian S, Guo Y (2001). Modulation of abscisic acid signal transduction and biosynthesis by an Sm-like protein in Arabidopsis.. Dev Cell.

[41] Gregory BD, O'Malley RC, Lister R, Urich MA, Tonti-Filippini J (2008). A link between RNA metabolism and silencing affecting Arabidopsis development.. Dev Cell.

[42] Verslues PE, Guo Y, Dong CH, Ma W, Zhu JK (2006). Mutation of SAD2, an importin beta-domain protein in Arabidopsis, alters abscisic acid sensitivity.. Plant J.

[43] Murguia JR, Belles JM, Serrano R (1996). The yeast HAL2 nucleotidase is an in vivo target of salt toxicity.. J Biol Chem.

[44] Davidian JC, Kopriva S (2010). Regulation of sulfate uptake and assimilation—the same or not the same?. Mol Plant.

[45] Leustek T, Saito K (1999). Sulfate transport and assimilation in plants.. Plant Physiol.

[46] Takahashi H, Kopriva S, Giordano M, Saito K, Hell R (2011). Sulfur assimilation in photosynthetic organisms: molecular functions and regulations of transporters and assimilatory enzymes.. Annu Rev Plant Biol.

[47] Mechold U, Ogryzko V, Ngo S, Danchin A (2006). Oligoribonuclease is a common downstream target of lithium-induced pAp accumulation in *Escherichia coli* and human cells.. Nucleic Acids Res.

[48] Zhu JK, Liu J, Xiong L (1998). Genetic analysis of salt tolerance in arabidopsis. Evidence for a critical role of potassium nutrition.. Plant Cell.

[49] Wilson PB, Estavillo GM, Field KJ, Pornsiriwong W, Carroll AJ (2009). The nucleotidase/phosphatase SAL1 is a negative regulator of drought tolerance in Arabidopsis.. Plant J.

[50] Hirsch J, Misson J, Crisp PA, David P, Bayle V (2011). A Novel *fry1* Allele Reveals the Existence of a Mutant Phenotype Unrelated to 5′->3′ Exoribonuclease (XRN) Activities in *Arabidopsis thaliana* Roots.. PLoS ONE.

[51] Renosto F, Patel HC, Martin RL, Thomassian C, Zimmerman G (1993). ATP sulfurylase from higher plants: kinetic and structural characterization of the chloroplast and cytosol enzymes from spinach leaf.. Arch Biochem Biophys.

[52] Lunn JE, Droux M, Martin J, Douce R (1990). Localization of ATP sulfurylase and O-acetylserine(thiol)lyase in spinach leaves.. Plant Physiol.

[53] Tyler JK (2002). Chromatin assembly. Cooperation between histone chaperones and ATP-dependent nucleosome remodeling machines.. Eur J Biochem.

[54] Kamiyama S, Sasaki N, Goda E, Ui-Tei K, Saigo K (2006). Molecular cloning and characterization of a novel 3′-phosphoadenosine 5′-phosphosulfate transporter, PAPST2.. J Biol Chem.

[55] Kamiyama S, Suda T, Ueda R, Suzuki M, Okubo R (2003). Molecular cloning and identification of 3′-phosphoadenosine 5′-phosphosulfate transporter.. J Biol Chem.

[56] Mandon EC, Milla ME, Kempner E, Hirschberg CB (1994). Purification of the Golgi adenosine 3′-phosphate 5′-phosphosulfate transporter, a homodimer within the membrane.. Proc Natl Acad Sci.

[57] Zaruba ME, Schwartz NB, Tennekoon GI (1988). Reconstitution of adenosine 3′-phosphate 5′-phosphosulfate transporter from rat brain.. Biochem Biophys Res Commun.

[58] Luders F, Segawa H, Stein D, Selva EM, Perrimon N (2003). Slalom encodes an adenosine 3′-phosphate 5′-phosphosulfate transporter essential for development in *Drosophila*.. EMBO J.

[59] Gazzani S, Lawrenson T, Woodward C, Headon D, Sablowski R (2004). A link between mRNA turnover and RNA interference in Arabidopsis.. Science.

[60] Cutler SR, Rodriguez PL, Finkelstein RR, Abrams SR (2010). Abscisic acid: emergence of a core signaling network.. Annu Rev Plant Biol.

[61] Fujii H, Chinnusamy V, Rodrigues A, Rubio S, Antoni R (2009). In vitro reconstitution of an abscisic acid signalling pathway.. Nature.

[62] Hauser F, Waadt R, Schroeder JI (2011). Evolution of abscisic acid synthesis and signaling mechanisms.. Curr Biol.

[63] Lee H, Xiong L, Ishitani M, Stevenson B, Zhu JK (1999). Cold-regulated gene expression and freezing tolerance in an *Arabidopsis thaliana* mutant.. Plant J.

[64] Chen H, Xiong L (2005). Pyridoxine is required for post-embryonic root development and tolerance to osmotic and oxidative stresses.. Plant J.

[65] Chen H, Xiong L (2010). *myo*-inositol-1-phosphate synthase is required for polar auxin transport and organ development.. J Biol Chem.

