# Supplementary figures and images for: A Nucleotide Metabolite Controls Stress-Responsive Gene Expression and Plant Development
Source: PLoS One. 2011 Oct 19;6(10):e26661. doi: 10.1371/journal.pone.0026661 (PMC3197580; doi:10.1371/journal.pone.0026661)

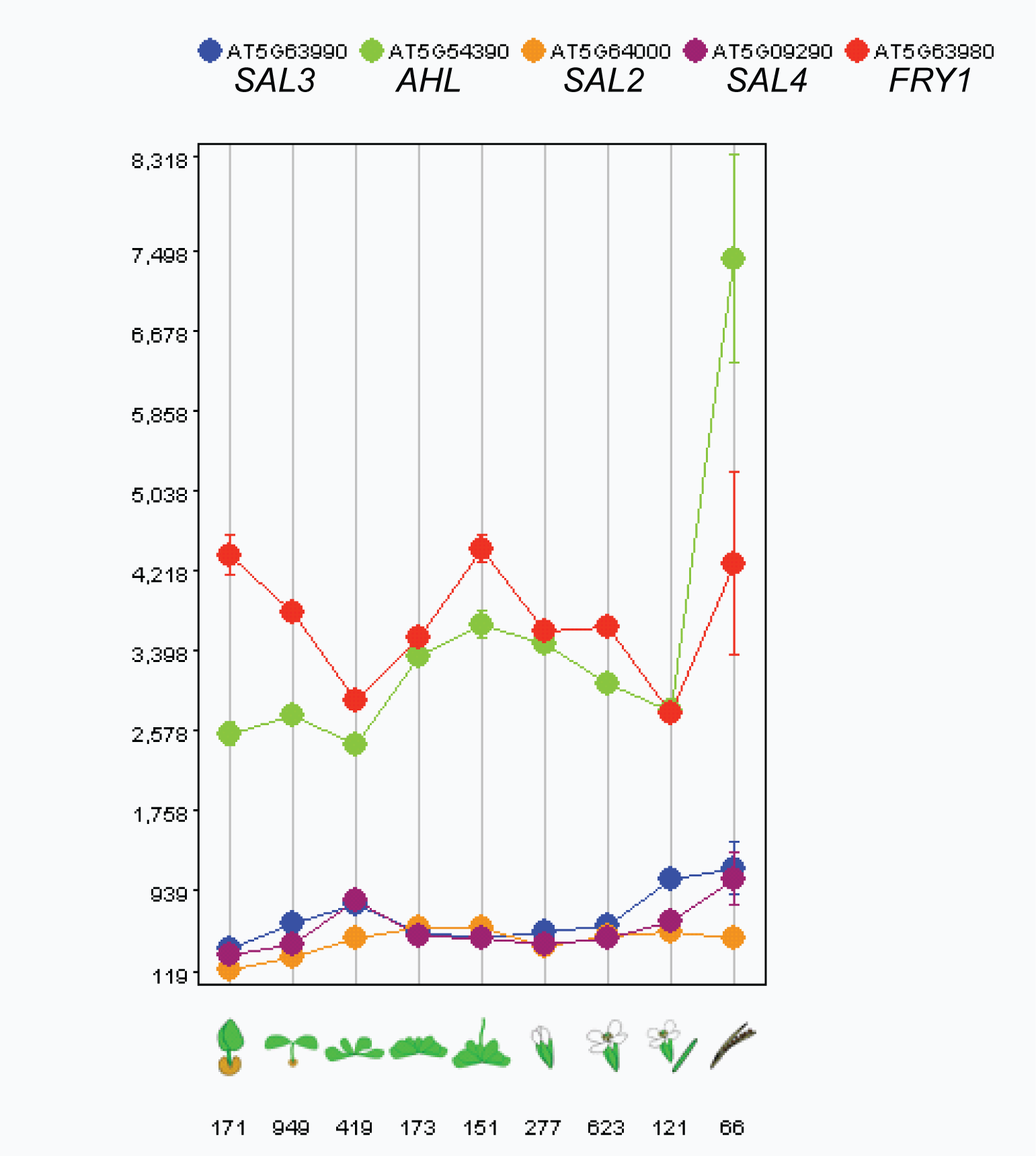

Supplement: Figure S1 — The transcript levels of FRY1 family members at different developmental stages. Data were extracted from the public microarray database (https://www.genevestigator.ethz.ch). (TIF) [file pone.0026661.s001.tif]

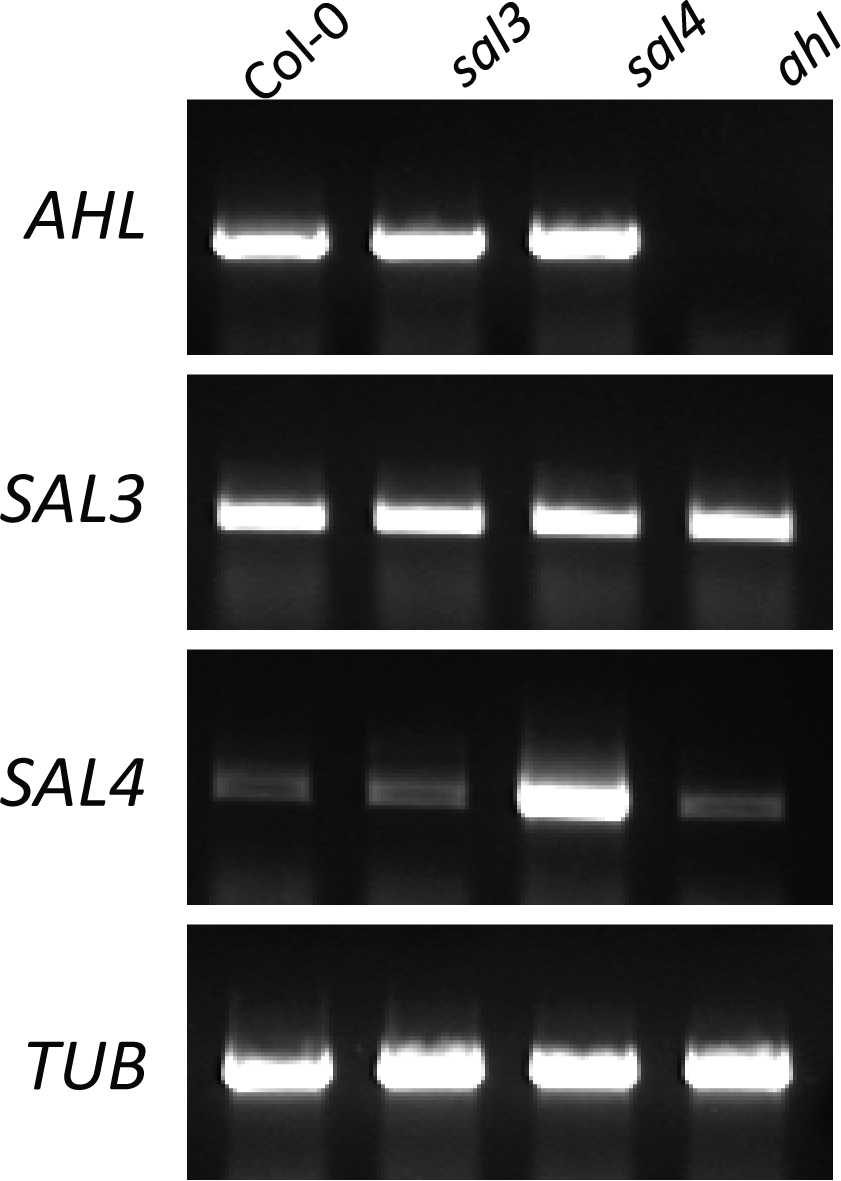

Supplement: Figure S2 — RT-PCR analysis of T-DNA insertion lines of FRY1 homologs. cDNA from T-DNA lines was amplified with primers specific to coding regions of FRY1 homologs; Col-0 is included as a positive control. (TIF) [file pone.0026661.s002.tif]

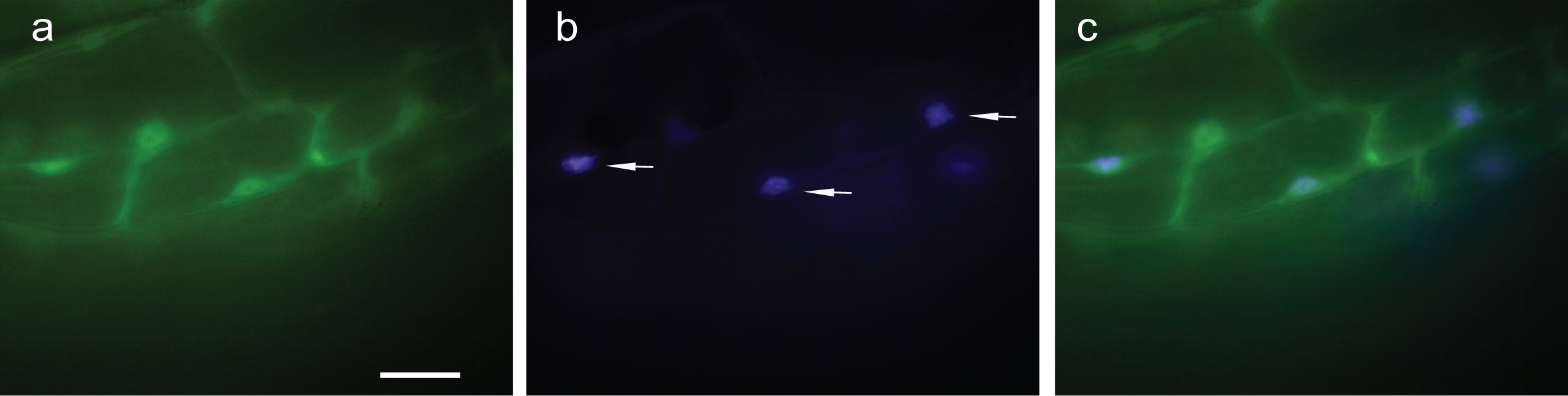

Supplement: Figure S3 — The subcellular localization of the FRY1ΔN54-GFP fusion protein in root epidermal cells. Green fluorescence from FRY1ΔN54-GFP (a) and DAPI fluorescence of stained nuclei (b) were monitored separately using an epifluorescence microscope (Nikon Eclipse E800). (c) is the merged images of (a) and (b). Bar = 50 µm. Arrow indicates nucleus in (b). (TIF) [file pone.0026661.s003.tif]

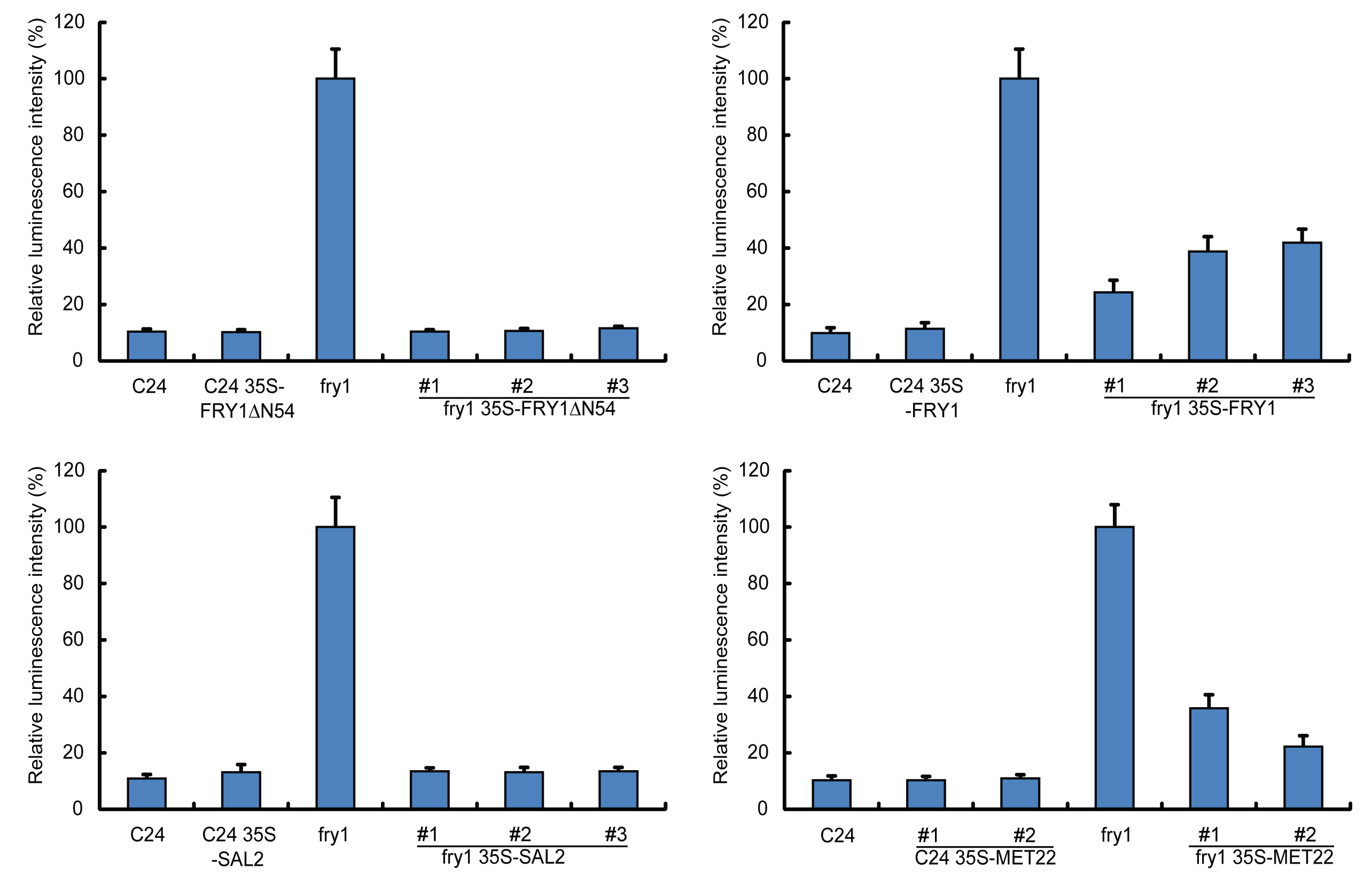

Supplement: Figure S4 — Relative luminescence intensity of seedlings in Figure 6. Data are means and SE from more than 25 seedlings. (TIF) [file pone.0026661.s004.tif]

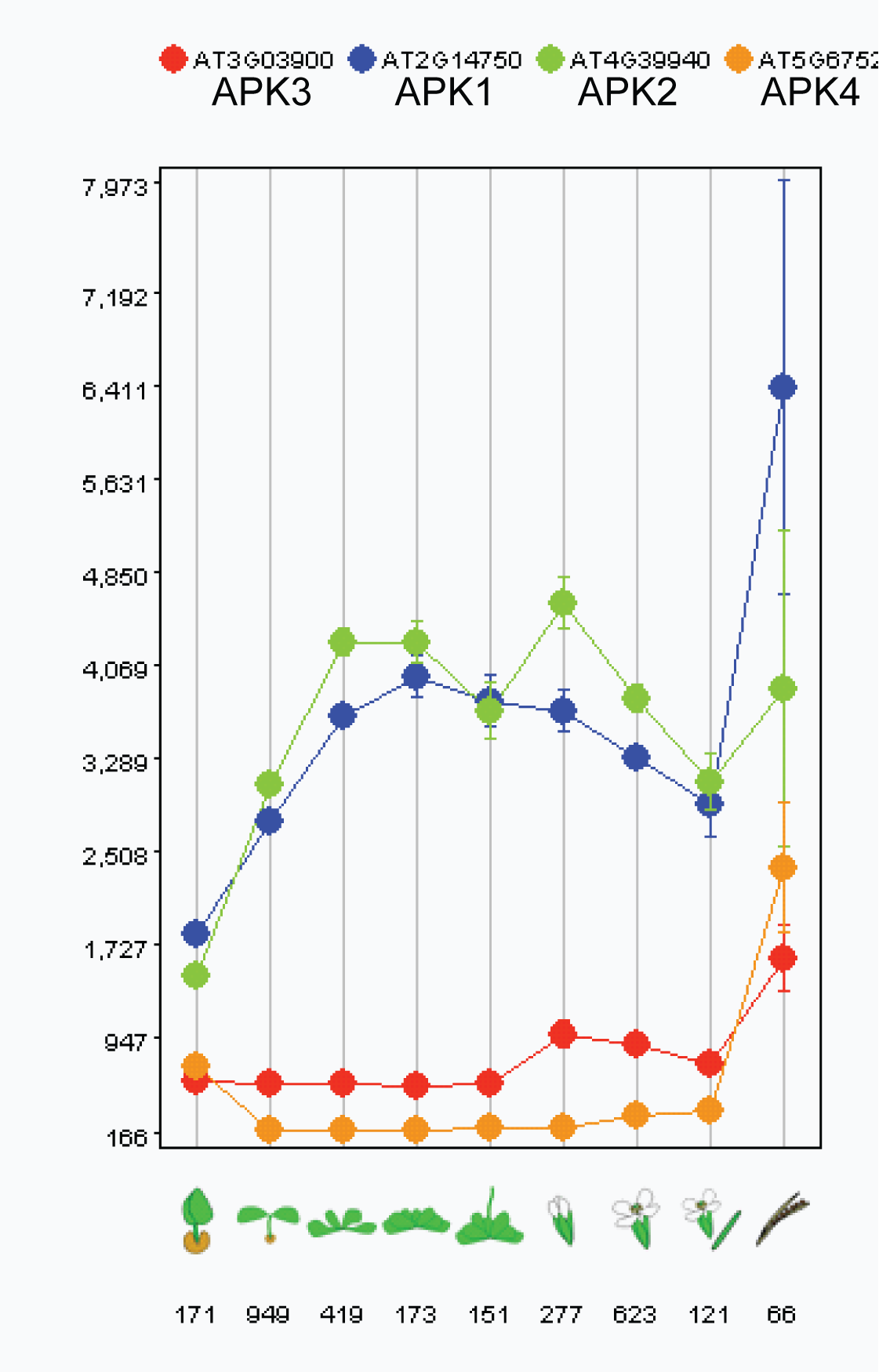

Supplement: Figure S5 — The transcript levels of APK family members at different developmental stages. Data were extracted from the public microarray database (https://www.genevestigator.ethz.ch). (TIF) [file pone.0026661.s005.tif]
